# Crohn’s Disease and Ulcerative Colitis Show Unique Cytokine Profiles

**DOI:** 10.7759/cureus.1177

**Published:** 2017-04-19

**Authors:** Zoltan H Nemeth, Dorian A Bogdanovski, Patricia Barratt-Stopper, Samantha R Paglinco, Luca Antonioli, Rolando H Rolandelli

**Affiliations:** 1 Department of Surgery, Morristown Medical Center; 2 Department of Clinical and Experimental Medicine, University of Pisa

**Keywords:** inflammation, crohn’s disease, ulcerative colitis, intestinal immunity, cytokine, chemokine, interleukin, polymerase chain reaction, inflammatory bowel disease (ibd)

## Abstract

**Introduction:**

Networks of cytokines have been implicated in both forms of inflammatory bowel disease (IBD): Crohn’s disease (CD) and ulcerative colitis (UC). While CD has associated with T-helper type 1 (Th1) immune responses, UC shows Th2 patterns. Recent studies reported that the inflamed intestinal regions in both CD and UC are significantly infiltrated with a newly described set of T helper, the Th17 cells. These cells have unique cytokine responses. These findings prompted us to further explore the cytokine profiles of CD and UC with a special focus on the Th2 and Th17 related mediators.

**Methods:**

Cytokine transcripts were compared using real-time polymerase chain reaction (PCR) in both inflamed and non-inflamed mucosal specimens from patients with active CD (n=35) or UC (n=20) and without CD or UC (Control, n=54).

**Results:**

In both CD and UC, interleukin (IL)-12 (p40), IL-18, IL-21 and IL-27 transcript levels were higher than in Control. The highest levels of cytokines were found in the diseased areas of CD and UC with only one exception; IL-12 (p40) in CD was more up-regulated in the non-diseased areas compared to diseased CD and Control specimens. CD samples but not UC specimens showed significant IL-17, IL-23, and IL-32 mRNA expression indicating a trend toward Th17 responses. In UC, however, IL-5, IL-13, IL-15 and IL-33 mRNA levels were significantly increased when compared to both CD and Control.

**Conclusions:**

The unique patterns of cytokine networks can help us to better understand the differential expression of their characteristic pathophysiology. In addition, the pharmacological regulation of these small molecules may hold promise to more effective and personalized therapies.

## Introduction

Ulcerative colitis (UC) and Crohn's disease (CD), the two major subsets of inflammatory bowel disease (IBD) are chronic and relapsing disorders leading to gastrointestinal damage [1-3]. The immune system mediates the pathogenesis of IBD as intestinal epithelial cells (IECs) recruit leukocytes to the gut mucosa [2-4]. The major hypothesis explaining IBD is that of a defect in the immune system's response to commensal microbiota, rather than a persistent pathogen [5]. Gut microbiota provides a constant, diverse source of antigens [3, 6-7-8]. The microbiota may break immune tolerance under some circumstances influenced by genetic susceptibility, lifestyle or environmental factors [2-4]. The proximity of gut-associated lymphoid tissue and luminal microbiota is likely to be responsible for these pathogenic stimuli [6-7, 9].

The respective roles of the innate and adaptive immune systems and their effector cells in IBD are an active area of research. For example, the particular antibodies to bacterial antigens present in IBD patients have been shown to yield useful diagnostic information, distinguishing the subtypes UC and CD [2, 7-8]. Furthermore, investigators are starting to elucidate the nature of disordered T-cell microbial recognition and effector function in IBD [2- 3-4, 8]. Studies of cytokines and transcription factors present in IBD patients have demonstrated that the Th1 and Th2 cell lineages are also characteristic in UC and CD, respectively [10-13]. More recent attention has focused on Th17 cells which develop from naive CD4+ T cells in response to IL-23 and send pro-inflammatory signals to other parts of the immune system using IL-17 [14-15]. The Th17 cells, native to the gut mucosal barrier, pathogenically change their behavior during IBD [14, 16]. The IL-23/IL-17 immune axis seems especially important in CD, where it may function as a parallel pathway to the Th1 response coordinating inflammation [17-20].

This work is a clinical study of cytokine signaling networks in IBD, using surgical bowel specimens from patients with or without UC and CD, and with or without an actively inflamed disease. We focused on lesser-studied pro- and anti-inflammatory cytokines associated with Th2 and Th17 differentiation and immunological behavior. A more detailed understanding of the cytokines involved in UC and CD may enable more personalized pharmacological therapies and advance understanding of the pathophysiology and the immunological differences between the two major IBD subtypes.

Although the entire pathophysiology of IBD is not fully understood, it has been shown that IBD is induced by an uncontrolled immune response to the intestinal content in patients with various genetic predispositions. One major group of regulatory and effector molecules of these immune responses are called cytokines and chemokines [21-24]. The delicate balance between the pro- and anti-inflammatory cytokines throughout the entire gastrointestinal tract is crucial for healthy intestinal barrier function and tissue homeostasis [20]. Changes in this balance, especially with pro-inflammatory cytokine overproduction can lead to active inflammation. The activation of various immune cells in inflamed tissue triggers a cascade of intracellular events which result in apoptosis, cellular infiltration and the loss of integrity and function of the gut. This is especially, the characteristic in the intestinal mucosal layer during the acute phases of IBD [25].

Networks of cytokines have been implicated in both Crohn’s disease (CD) and ulcerative colitis (UC). While CD is primarily associated with T-helper type 1 (Th1) immune responses, UC predominantly shows a characteristic atypical Th2 cytokine pattern [20]. Recent studies, however, reported that the inflamed intestinal regions in both CD and UC are significantly infiltrated with a newly described set of T helper, the Th17 cells with unique cytokine responses [14-15, 26]. However, the exact network and pathophysiology of these cytokines in the development of both diseases are not yet completely elucidated. This prompted us to further explore the cytokine profiles of CD and UC with a special focus on the Th2 and Th17 related mediators [14-15, 26]. We wanted to establish cytokine patterns which can correspond with the disease activity and inflammation intensity seen in IBD. We also attempted to better characterize the cytokine profiles of the two main types of IBD which can reflect on their respective activity and differentiate between them. This is especially important as observing and regulating cytokine expression and function in both forms of IBD is a promising approach that may lead us to develop more effective and personalized pharmacological therapies.

## Materials and methods

### Subjects of the study

This study was approved by the Institutional Review Board (IRB) of the Atlantic Health system. Patients provided written informed consent prior to undergoing surgery. Surgical bowel specimens were collected from patients with or without IBD who underwent a colorectal surgical procedure at the department of surgery at Morristown Medical Center. Altogether, samples from 112 patients were analyzed in 2010, 2011 and 2012. In Table 1 patients’ demographic and clinical data are given.

**Table 1:** Patients’ demographic and clinical characteristics

	Non-IBD	CD	UC
Number of patients	54	35	20
Age (Years, Mean ± SEM)	58.4 ± 1.5	37.2 ± 3.9	42.6 ± 5.7
Sex (F : M)	26 : 28	15 : 20	12 : 8

A total of four intestinal specimens were collected from each patient with IBD, two from the inflamed areas and two from nearby but macroscopically non-inflamed areas. Five study groups were composed as follows: Control or "Non-IBD" (Non-IBD); Crohn’s Disease with the un-inflamed area (CD-N); Crohn’s Disease with inflamed area (CD-I); ulcerative colitis with the un-inflamed area (UC-N); and ulcerative colitis with inflamed area (UC-I).

### Preparation of experimental samples

Intestinal tissue samples were taken immediately after the segment of intestine was removed. The samples were submerged in one ml of ribonucleic acid RNA-Later solution (Ambion, Austin, Texas, USA) and stored at -20 °C until processing. Protein and total ribonucleic acid (RNA) were isolated from specimens using Tri-Reagent (Molecular Research Center, Inc., Cincinnati, OH, USA) following manufacturer’s instructions. Protein and RNA concentration were measured using BMG Labtec FLUOstar OPTIMA microplate reader (BMG LABTECH Inc., Cary, NC, USA).

### Quantitative polymerase chain reaction

Quantification of the expression level changes of target mRNA-s in the collected intestinal samples was performed using real-time PCR method. First, we carried out the reverse transcription of the mRNA using high capacity cDNA Reverse Transcription Kit (Applied Biosystems, Foster City, CA, USA) starting with 0.5 μg RNA. Then real-time PCR was performed using SYBR Green Master Mix, with cDNA and specific primer sets for our target genes. Expression levels of transcripts were compared in these five groups: CD-N, CD-I, No-IBD, UC-N and UC-I. Cytokine primer sets are shown in Table 2. The Applied Biosystems 7700 (Applied Biosystems, Foster City, CA, USA) sequence detector was used for amplification of target sequences and quantitation of differences between treatment groups was done using the comparative threshold cycle (CT) method.

**Table 2:** List of primers used in real-time PCR studies

Target gene	forward (5'-3') primer	reverse (5'-3') primer
IL-32	TGA GGA GCA GCA CCC AGA GC	CCG TAG GAC TGG AAA GAG GA
IL-23 (p19)	AGC AGC TCA AGG ATG GCA CTC AG	CCC CAA ATT TCC CTT CCC ATC TA
IL-27 (p28)	GCG GAA TCT CAC CTG CCA	GGA AAC ATC AGG GAG CTG CTC
IL-15	TGT CTT CAT TTT GGG CTG TTT CA	TCC TCC AGT TCC TCA CAT TCT TTG
IL-18	GCT TGA ATC TAA ATT ATC AGT C	CAA ATT GCA TCT TAT TAT CAT G
IL-33	TGA GTC TCA ACA CCC CTC AAA TG	GGC ATG CAA CCA GAA GTC TTT T
IL-5	AAG AGA CCT TGG CAC TGC TTT C	GGA ACA GGA ATC CTC AGA GTC TCA
IL-13	ACA GCT GGC ATG TAC TGT GC	CAA CTT TCT ATT ATC CAC TC
IL-12 (p40)	AAG GAA GAT GGA ATT TGG TCC ACT CCA CTC	GAT GTC CCT GAT GAT GAA GAA GCT G
IL-21	TGT GAA TGA CTT GGT CCC TGA A	AAC AGG AAA AAG CTG ACC ACT CA
IL-17	AAT CTC CAC CGC AAT GAG GA	ACG TTC CCA TCA GCG TTG A
β-actin	TGC CGA CAG GAT GCA GAA G	GCC GAT CCA CAC GGA GTA CT
18S	CTT AGA GGG ACA AGT GGC G	ACG CTG AGC CAG TCA GTG TA

### Statistical analysis

Triplicate PCR samples were analyzed and the results obtained as a ratio cytokine/β-actin mRNA levels were expressed as arbitrary units. Results are expressed as means ± standard error of the mean (SEM) of 'n' observations. Statistical differences in cytokine mRNA expression levels between these five groups: CD-N, CD-I, non-IBD, UC-N and UC-I were analyzed using the t-test or the Mann-Whitney U-test. A value of p < 0.05 was considered statistically significant; * p < 0.05 versus C;** p < 0.01 versus C; *** p < 0.001 versus No-IBD. Statistical analysis was performed using GraphPad Prism 5 software (GraphPad Software, Inc., La Jolla, CA, USA).

## Results

### Both CD and UC specimens display upregulated transcript levels of IL-12, IL-18, Il-21 and IL-27 in both inflamed and non-inflamed intestinal areas when compared to non-IBD

**Figure 1:**
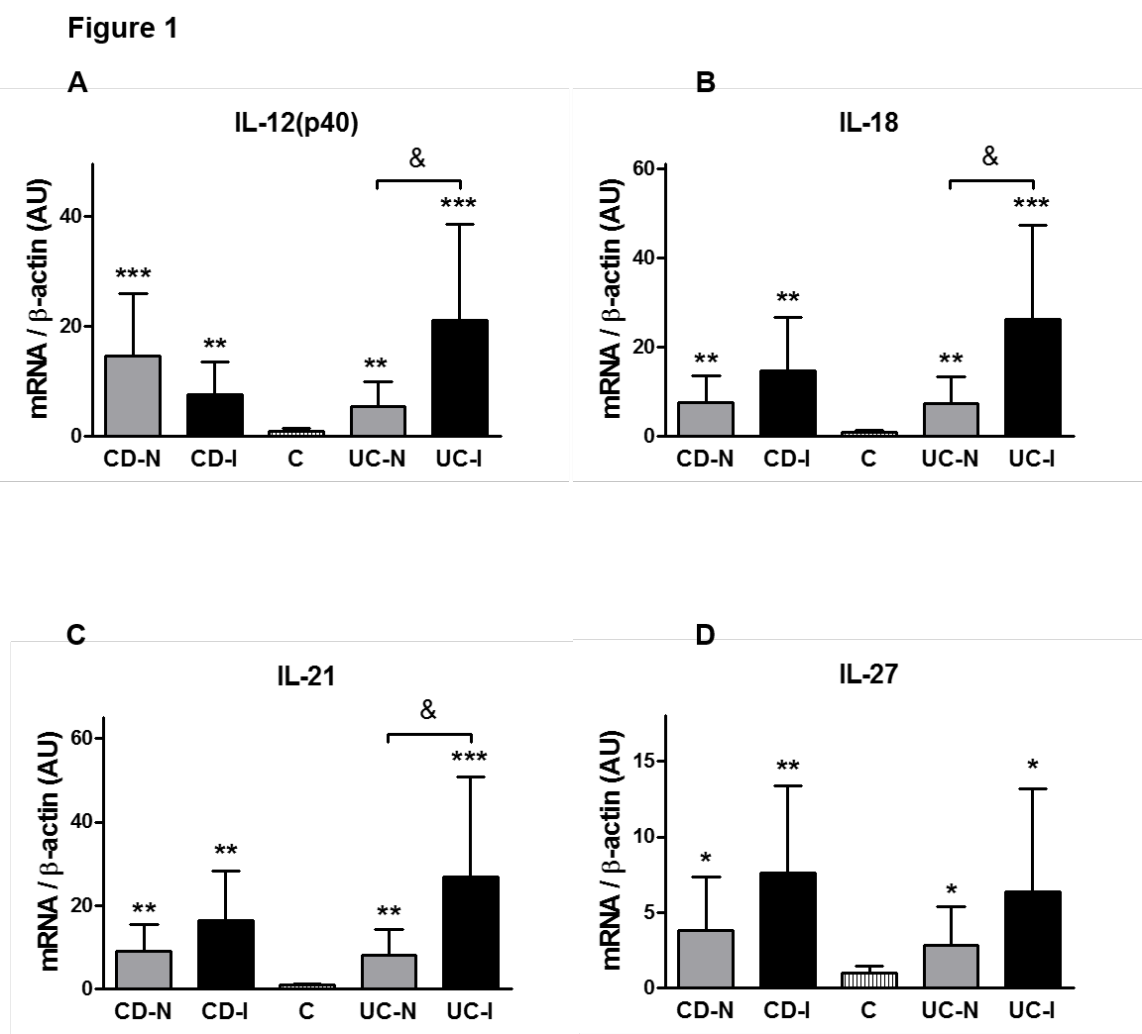
In both CD and UC, interleukin (IL)-12 (p40), IL-18, IL-21 and IL-27 transcript levels were higher than in the Control (C) group

We investigated the relative mRNA levels of these cytokine transcripts in both inflamed and non-inflamed surgical bowel mucosal specimens from patients with active CD (n=35) or UC (n=20) as well as intestinal samples, from patients without IBD (Figure 1). We found that interleukin (IL)-12 (p40), IL-18, IL-21 and IL-27 transcript levels were elevated in both CD and UC patients relative to the non-IBD specimens. In addition, in UC patients the inflamed bowel specimens had significantly higher levels of IL-12 (p40), IL-18, and IL-21, but not IL-27, than the non-inflamed areas. In contrast, when comparing inflamed and non-inflamed specimens from CD patients, we could not detect this distinction between diseased and non-diseased CD specimens when tested for the above cytokine transcripts (Figure 1).

### CD samples but not UC specimens show significant IL-17, IL-23, and IL-32 mRNA expression levels in comparison to non-IBD

**Figure 2:**
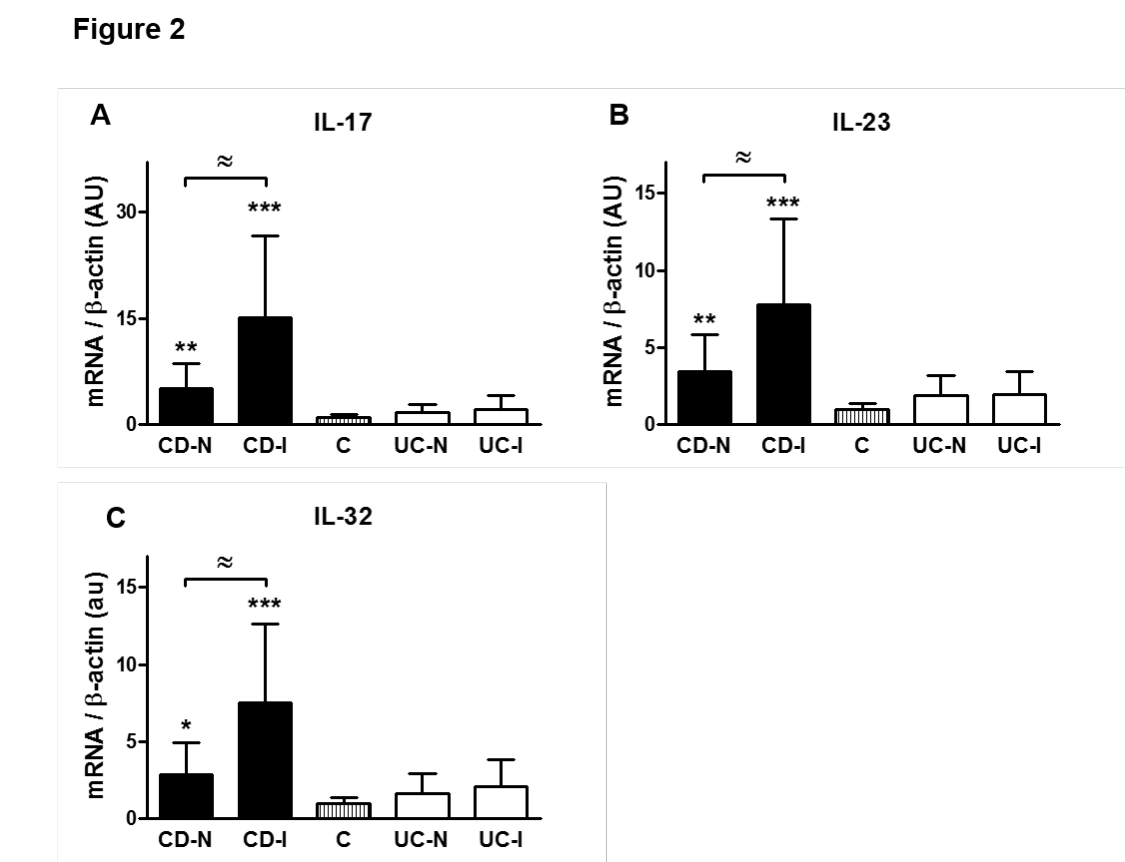
CD samples but not UC specimens showed significant IL-17, IL-23, and IL-32 mRNA expression levels indicating a tilt toward Th17-type responses

We also measured the relative expression characteristics of the mRNA of the above molecules in specimens from the same patient groups and found that expression of IL-17, IL-23, and IL-32 was significantly elevated only in patients with CD in their inflamed segment when compared to non-IBD (Figure 2). Furthermore, within the CD group, the transcripts of all these three cytokines were higher in the inflamed areas as compared to un-inflamed CD bowel samples (Figure 2).

### Specimens from UC patients only (and not from CD) have increased IL-5, IL-13, IL-15, and IL-33 mRNA expression levels compared to non-IBD samples

**Figure 3:**
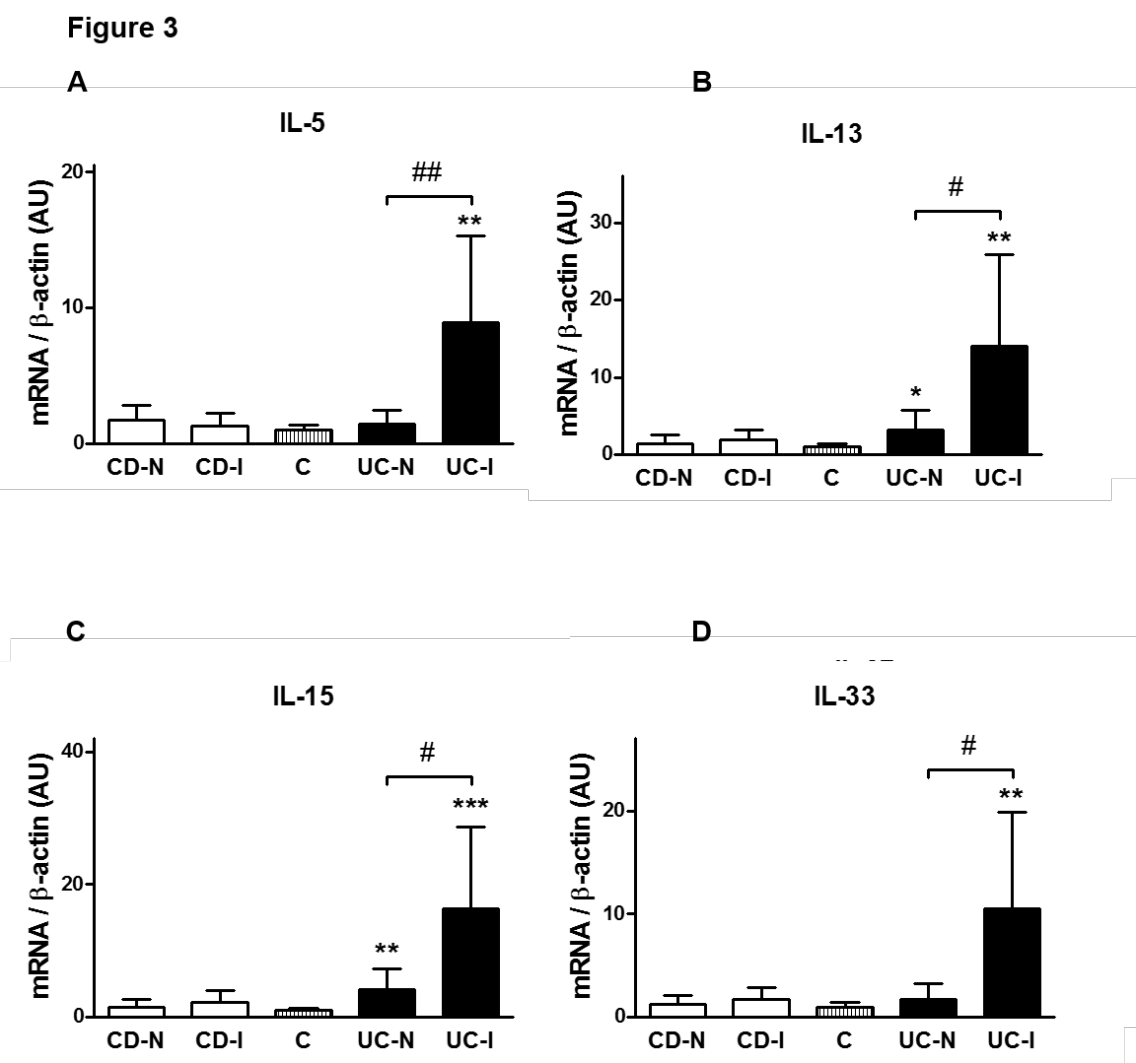
In UC, IL-5, IL-13, IL-15 and IL-33 mRNA levels were significantly increased when compared to both CD and Control

In addition, we assessed the transcript expression of IL-5, IL-13, IL-15, and IL-33 in the same five groups of intestinal samples: C or Non-IBD; CD-N; CD-I; UC-N; and UC-I. We were able to detect that only UC samples expressed increased transcript levels for IL-5, IL-13, IL-15, and IL-33 in comparison to non-IBD (Figure 3). Moreover, in this cytokine panel we documented a significant upregulation of all four molecules when comparing inflamed tissue samples to non-inflamed ones within the UC patient group (Figure 3).

## Discussion

Our findings show that several cytokines were significantly upregulated in both forms of IBD (CD and UC) when compared to the non-IBD or Control group. In our study, these cytokines include interleukin (IL)-12 (p40), IL-18, IL-21, and IL-27. We found that transcript levels of IL-12 were elevated in both subtypes of IBD and that level of the structurally related (via the IL-12p40 subunit) IL-23 was elevated in CD but not in UC [22]. The unequal elevation of IL-23 in CD samples supports the general hypothesis that CD is characterized by a Th1 driven response. The IL-23 mediated pathways are important in Th17 development and expansion subsequent to Th1 activation [26]. As UC is driven by a Th2 mediated response, it follows that IL-23 was not found to be elevated in the UC samples. Apilimod mesylate (STA-5326), a small molecule inhibitor of IL-12 and IL-23 through the shared p40 epitope, was successful in Phase I/IIa clinical trials in patients with active CD, showing that the specific relationship between the IL-12 and IL-23 cytokine family and CD is well-established [27-28].

More interestingly, the transcript levels of IL-12, IL-18, IL-21, and IL-27 were also significantly elevated in the non-inflamed areas of the bowel of patients with CD or UC. This observation may clearly have future diagnostic value when analyzing biopsy specimens from macroscopically healthy looking areas. It has been published that CD and UC can display an increased immune activity reflected by higher levels of immune mediators even in areas of the intestine without histological signs of inflammation. This is the case, for example, with TNF-α, IFN-γ, and IL-6 in both CD and UC [29-30].

Our additional data confirms that CD specimens but not UC specimens showed significant IL-17, IL-23, and IL-32 mRNA expression levels indicating a tendency toward Th17-type responses. This observation can have diagnostic value in determining accurate diagnosis and treatment options in clinical cases of IBD. In agreement with this finding, it has been shown that patients with CD have higher CD161+ cells in both their blood and colonic mucosa. It is interesting to note that these CD161+ cells secrete high quantities of IFN-γ and IL-17. Moreover, CD patients show higher IL-23-receptor (IL-23R) expressing T cells that are able to produce IFN-γ, IL-17 and IL-22 when stimulated with circulating IL-23 [27-28]. These findings clearly identify IFN-γ and IL-17 produced by Th17 cells as critical mediators of active inflammation in CD.

Furthermore, we also found that in UC specimens IL-5, IL-13, IL-15 and IL-33 mRNA levels were significantly increased when compared to both CD and Control. The relationship between IL-5 and IL-13 with Th2 polarization is well-established [31] so their presence in UC patients supports the hypothesis that UC is a generally Th2-driven disease. The same is true for IL-33, a more recently characterized cytokine which induces Th2 cytokines IL-4, IL-5, and IL-13 from T cells and plays a role in several chronic inflammatory disorders, including UC [32]. There is a paucity of previous research available relating IL-15 to IBD although it is well known that IL-15 regulates T-cell homeostasis.

Supporting this interpretation, we also detected that the characteristic Th17 cytokine IL-17 was elevated in patients with UC only. This cytokine and its subtypes IL-17A-F exert diverse and ambiguously pro- and anti-inflammatory effects on IBD in animal models [4]. We found that IL-21 was elevated in both patients with CD and UC. IL-21 has a well-established role in Th17 function, suppressing the expansion of immunosuppressive T regulatory cells and thus promising a prolonged inflammatory response [33]. IL-21 also signals back to Th1 cells to increase inflammatory interferon gamma (IFN-gamma) production, suggesting a positive feedback loop. But although IL-21's functions have been typically understood to be associated with a predominant Th1 cell response, our data shows IL-21 mRNA elevated in both IBD subtypes, rather than in CD only. This is consistent with a recent study of human mucosal samples [33].

Transcripts of IL-32 were found to be elevated in patients with CD and not UC. IL-32 is induced by Th1 cytokines such as IL-12 and IFN-gamma and seems to amplify innate immune response through a nucleotide-binding oligomerization domain proteins (NOD2)-dependant pathway [33]. This data, therefore, supports the view of CD as a Th1-driven disease.

The cytokines IL-5, IL-13, IL-15, and IL-33 were elevated in UC and not CD and considerably more elevated in patients with active disease than those without.

Altogether, we documented marked differences in the distribution of several cytokines among IBD subtypes. The significance of these results lies primarily in the effort to establish a reliable and efficient differential diagnosis of IBD subtypes and secondarily, to develop more effective and personalized therapies for IBD.

Inflammatory cytokines such as those investigated in this study are potential drug targets and understanding their roles and relationships with IBD may lead to more effective and more personalized therapies. Currently one of the most effective biologic treatments for IBD is infliximab, an antibody targeting tumor necrosis factor (TNF-alpha) [4, 34]. Genetic research suggests that TNF-alpha may operate at a bottleneck for the pathogenesis of both IBD subtypes. Other neutralizing antibodies targeting inflammatory cytokines may need to be more personalized. Therefore, this experiment's exploration of the distribution of these potential drug targets among IBD subtypes, especially those with a less well-established role in IBD such as IL-32 and the Th2-associated cytokines (IL-5, IL-13, IL-15, IL-33) offers insight into the design of studies developing the pharmacological intervention for IBD in the future.

Our findings point towards those cytokines which are most characteristic of the UC or CD disease states, with the caveat that, elevation of those cytokines with the most potential to establish a diagnosis of UC or CD show only mild elevation as compared to patients without active disease. These results are comparable to previous studies which established a set of inflammatory markers currently used to differentiate IBD subtypes [35-39]. Further research will demonstrate to what extent the transcription levels of these cytokines will aid in diagnosing IBD, alongside already established methods of clinical testing.

## Conclusions

In this study, it was found that in both CD and UC, the transcription levels of IL-12, IL-18, IL-21, and IL-27 were higher than in normal tissue. IL-12 was found to have a higher transcription level in the non-diseased areas of CD samples than in the diseased areas of CD samples and Control specimens. IL-17, IL-23, and IL-32 mRNA had significantly higher levels of expression in CD specimens than in UC specimens, indicating a trend towards Th17 responses. The IL-5, IL-13, IL-15 and IL-33 expression are significantly higher in UC specimens than in both CD and Control specimens.

This study shows that specimens with UC and CD each show their own independent cytokine profiles. Understanding these cytokine networks can help healthcare providers better understand the differential expression of these diseases' characteristic pathophysiologies. With this knowledge, more sensitive and specific diagnostic tests may be developed, as well as personalized therapies which may effectively treat individuals based on their own physiological needs.
